# Development and Validation of Machine Learning Models to Predict Postoperative Delirium Using Clinical Features and Polysomnography Variables

**DOI:** 10.3390/jcm13185485

**Published:** 2024-09-16

**Authors:** Woo-Seok Ha, Bo-Kyu Choi, Jungyeon Yeom, Seungwon Song, Soomi Cho, Min-Kyung Chu, Won-Joo Kim, Kyoung Heo, Kyung-Min Kim

**Affiliations:** 1 Department of Neurology, Severance Hospital, Yonsei University College of Medicine, Seoul 03722, Republic of Korea; 2 Department of Biomedical Systems Informatics, Yonsei University College of Medicine, Seoul 03722, Republic of Korea; 3 Department of Neurology, Gangnam Severance Hospital, Yonsei University College of Medicine, Seoul 06273, Republic of Korea

**Keywords:** postoperative delirium, machine learning, polysomnography, sleep disorders, predictive modeling

## Abstract

**Background:** Delirium affects up to 50% of patients following high-risk surgeries and is associated with poor long-term prognosis. This study employed machine learning to predict delirium using polysomnography (PSG) and sleep-disorder questionnaire data, and aimed to identify key sleep-related factors for improved interventions and patient outcomes. **Methods:** We studied 912 adults who underwent surgery under general anesthesia at a tertiary hospital (2013–2024) and had PSG within 5 years of surgery. Delirium was assessed via clinical diagnoses, antipsychotic prescriptions, and psychiatric consultations within 14 days postoperatively. Sleep-related data were collected using PSG and questionnaires. Machine learning predictions were performed to identify postoperative delirium, focusing on model accuracy and feature importance. **Results:** This study divided the 912 patients into an internal training set (700) and an external test set (212). Univariate analysis identified significant delirium risk factors: midazolam use, prolonged surgery duration, and hypoalbuminemia. Sleep-related variables such as fewer rapid eye movement (REM) episodes and higher daytime sleepiness were also linked to delirium. An extreme gradient-boosting-based classification task achieved an AUC of 0.81 with clinical variables, 0.60 with PSG data alone, and 0.84 with both, demonstrating the added value of PSG data. Analysis of Shapley additive explanations values highlighted important predictors: surgery duration, age, midazolam use, PSG-derived oxygen saturation nadir, periodic limb movement index, and REM episodes, demonstrating the relationship between sleep patterns and the risk of delirium. **Conclusions:** The artificial intelligence model integrates clinical and sleep variables and reliably identifies postoperative delirium, demonstrating that sleep-related factors contribute to its identification. Predicting patients at high risk of developing postoperative delirium and closely monitoring them could reduce the costs and complications associated with delirium.

## 1. Introduction

Delirium is prevalent among hospitalized patients and is characterized by acute alterations in consciousness that manifest as changes in concentration and orientation, and hallucinations. Postoperative delirium is one of the most frequent surgical complications, with occurrences ranging from 15% to 25% in patients undergoing major elective surgeries and up to 50% in those undergoing high-risk procedures, such as hip or heart surgeries [1,2,3]. Delirium not only contributes to increased mortality rates but also extends hospital stays and increases the risk of long-term dementia [4,5,6]. Consequently, predicting which patients are likely to develop postoperative delirium can help focus monitoring resources and reduce both complications and costs.

Sleep disorders and delirium share many common symptoms and mechanisms. For instance, severe sleep deprivation can lead to perception abnormalities, aggressive behavior, hallucinations, and concentration difficulties, mirroring delirium symptoms [7]. Electrophysiological studies have shown that delirium is associated with a rapid decrease in rapid eye movement (REM) sleep, linking it to alterations in sleep architecture [8,9]. Imbalances in acetylcholine and melatonin levels have been implicated in both sleep disturbance and delirium [10]. A recent meta-analysis identified several predisposing factors, including OSA (obstructive sleep apnea) and poor sleep quality, that are significantly associated with delirium, in addition to well-known risk factors like older age, prolonged surgery time, and intravenous anesthesia [2]. Additionally, the inclusion of sleep disturbances in the symptom criteria for diagnosing delirium in the Diagnostic and Statistical Manual of Mental Disorders, 5th Edition has heightened awareness of the connection between sleep and delirium [11].

In this context, several studies have investigated the association between sleep-related variables and delirium and have attempted to predict postoperative delirium using these variables. Some studies demonstrated that poor sleep quality is associated with an increased risk of delirium, using the Pittsburgh Sleep Quality Index (PSQI) [12,13]. Actigraphy studies have shown that shorter preoperative sleep duration increases the risk of postoperative delirium [14]. Research utilizing polysomnography (PSG) variables such as sleep-disordered breathing, total sleep time, and sleep stage distribution have further illustrated this connection [15,16,17]. However, these studies were limited by small sample sizes and did not combine clinical and sleep variables to develop predictive models for individual delirium risk.

This study aimed to investigate the factors associated with postoperative delirium by integrating clinical variables related to delirium with sleep variables obtained using PSG. By combining these data, we aimed to develop a machine learning model to predict postoperative delirium at the individual level, validate its performance, and identify the key variables that explain the model.

## 2. Materials and Methods

### 2.1. Data and Participants

**Figure 1:**
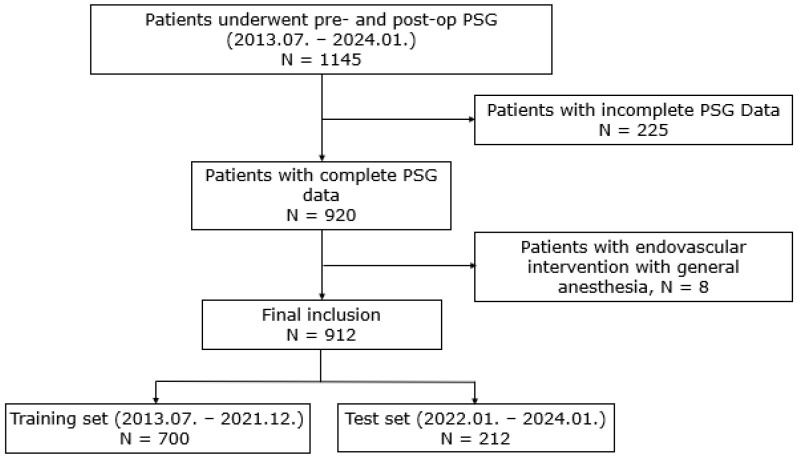
A flowchart depicting the participants in this study.

The participants in this study were enrolled as illustrated in Figure 1. We retrospectively collected information related to the participants, who underwent surgery under general anesthesia at the Severance Hospital between July 2013 and January 2024. The inclusion criteria were as follows: (1) aged ≥ 18 years and (2) underwent PSG within the 5 years before or after the surgery at the same center. Considering that the aim of this study was to examine the association with delirium, we excluded pediatric patients. A total of 1145 adult patients were included. After excluding 225 patients with insufficient PSG data and 8 who underwent intravascular intervention under general anesthesia, 912 patients were finally enrolled (Figure 1). Patients who underwent surgery up to December 2021 were assigned to the training set (700 patients), and those who underwent surgery from January 2022 onwards were assigned to the test set (212 patients).

### 2.2. Delirium Assessments

The diagnosis of delirium was established if a new diagnosis related to delirium (International Classification of Diseases 10th edition codes F05.9 [Delirium], F05.8 [Delirium due to multiple etiologies], and F05.1 [Delirium superimposed on dementia]) was recorded during the same hospitalization as the surgery, if new antipsychotic medications commonly used for the acute treatment of delirium (haloperidol, olanzapine, quetiapine, risperidone, or ziprasidone) were issued within 14 days postoperatively, or if a psychiatric consultation related to delirium was conducted within 14 days postoperatively.

### 2.3. Clinical Variables

Clinical variables collected from the patients included age, sex, height, weight, and underlying conditions (hypertension [HTN], diabetes mellitus [DM], cardiac disease, and brain disease). Surgery-related variables included the American Society of Anesthesiologists class, anesthetic agents used, type of surgery, duration of surgery, and emergency-surgery status. Laboratory results were also collected, including hemoglobin and platelet count, and albumin, aspartate aminotransferase (AST)/alanine aminotransferase (ALT), creatinine, sodium, and potassium levels. These were assessed to determine whether they fell outside the normal range, and categorized as anemia, thrombocytopenia, hypoalbuminemia, AST/ALT elevation, creatinine elevation, hyponatremia, and hypokalemia, respectively.

### 2.4. PSG

We conducted overnight in-laboratory PSG recordings using Natus SleepWorks Software (Natus Medical Inc., Pleasanton, CA, USA). Participants were instructed to sleep in a controlled environment with dim lighting, temperature regulation, and noise control. The PSG recordings included electroencephalography with frontal, central, and occipital electrodes; 1-lead electrocardiography; electromyography of extraocular eye movement, chin, and bilateral anterior tibialis muscles; nasal airflow and thermistor; peripheral oxygen saturation; sleep position; and chest and abdominal plethysmography. Sleep staging and scoring for respiratory events and movements followed the guidelines of the American Academy of Sleep Medicine and were conducted by two sleep technicians with >10 years of experience [18,19]. Three neurologists (WSH, SC, and KMK), experienced in PSG interpretation and responsible for training residents in this field, meticulously reviewed all PSG data. The sleep parameters derived from the PSG included time in bed (TIB), total sleep time, wake after sleep onset, sleep latency, sleep efficiency, percentage of each sleep stage (N1, N2, N3, and REM) relative to total sleep, number of REM episodes, REM sleep latency, number of awakenings during sleep, arousal index, apnea–hypopnea index (AHI), minimum oxygen saturation, snoring, periodic limb movements (PLMs), and the PLMs with arousal (PLMar) index.

### 2.5. Sleep Questionnaires

Questionnaires, including the PSQI, Insomnia Severity Index (ISI), Epworth Sleepiness Scale (ESS), STOP-Bang score, and Berlin questionnaire, were administered to assess participants’ sleep habits and related problems before PSG. The PSQI assesses sleep quality and disturbances on a scale ranging from 0 to 21, considering aspects such as sleep duration, efficiency, interruptions, and daytime dysfunction over one month [20]. The ISI is a seven-item questionnaire measuring insomnia severity over the previous two weeks, with scores ranging from 0 to 28. It assesses various aspects of sleep disruption and its impact on daily life [21]. The ESS is an eight-item questionnaire evaluating daytime sleepiness. Participants rated their likelihood of dozing off in different situations, with scores ranging from 0 to 24 [22]. The STOP-Bang questionnaire scores individuals on eight factors, ranging from 0 to 8, including snoring, tiredness, observed apnea, high blood pressure, body mass index (BMI), age (≥50 years), neck circumference, and male gender [23]. Higher scores indicate a higher risk of obstructive sleep apnea. The Berlin questionnaire evaluates the risk of sleep apnea through snoring, daytime fatigue, and HTN/obesity. It categorizes individuals as high or low risk [24].

### 2.6. Machine Learning

The dataset used in this study was derived from the clinical records of patients undergoing surgical procedures and was formatted and anonymized prior to analysis. After the initial cleaning steps, which included removing duplicates and handling missing values through imputation, the data were split into training and test sets. This split was used to train and evaluate the machine learning models described below.

We employed six machine learning algorithms (decision tree, Random Forest, extreme gradient boosting [XGBoost], light gradient-boosting machine [LightGBM], support vector machine [SVM], and artificial neural network [ANN]) to predict the occurrence of delirium post-surgery. These algorithms were selected for their ability to capture the complex factors contributing to delirium. Decision tree and Random Forest models were chosen for their interpretability and ability to enhance prediction accuracy. XGBoost and LightGBM were utilized for their efficiency in handling large datasets and identifying subtle patterns. SVM was employed to explore both linear and non-linear relationships, while ANN was used to model complex, non-linear interactions between clinical and sleep variables. Each model was trained on a training set with hyperparameters tuned using a grid search and cross-validation of the training data. The performance of the models was evaluated using the testing set based on accuracy and area under the receiver operating characteristic curve (AUROC). Accuracy measures the overall correctness of the model, precision assesses the proportion of true positives among the predicted positives, recall evaluates the model’s ability to identify all relevant instances, and weighted F1-score provides a harmonic mean of precision and recall, offering a balanced evaluation of the model’s performance.

A decision tree classifier was implemented using the scikit-learn library, with the tree depth restricted to prevent overfitting, and the maximum depth was set through cross-validation. The Random Forest model, also implemented in scikit-learn, consists of an ensemble of decision trees with parameters such as the number of trees (n_estimators) and the maximum depth of each tree (max_depth), optimized via cross-validation. XGBoost was used to train a gradient-boosting model that minimized a regularized objective function with important hyperparameters, including n_estimators, max_depth, learning_rate, and subsample rates tuned to optimize the performance. LightGBM, another gradient-boosting framework, was chosen for its efficiency with large datasets, and parameters such as num_leaves, max_depth, learning_rate, and n_estimators were tuned to determine the optimal settings. The SVM model was used to classify data by finding the optimal hyperplane that maximized the margin between classes. A kernel function was applied to handle nonlinear relationships in the data. An ANN model with an input layer, hidden layers, and output layer was used. Neurons applied weighted sums and nonlinear activation functions, and the model was trained using backpropagation and gradient descent to minimize the loss function.

Shapley additive explanations (SHAP) were adopted to verify the explainability of the artificial intelligence (AI) models [25,26]. These values highlighted the most influential factors in predicting delirium and provided insights into the underlying patterns recognized by the models.

### 2.7. Statistical Analyses

All statistical analyses were conducted using Python (Python Software Foundation, Wilmington, DE, USA). Continuous variables were analyzed using the Student’s t-test for normally distributed data and the Mann–Whitney U test for non-parametric data. Categorical variables were compared using Pearson’s χ^2^ test or Fisher’s exact test. The primary outcome for evaluating the model’s performance was AUROC, with additional metrics including accuracy, precision, recall, and weighted F1-score also being assessed. Statistical significance for all analyses was set at *p* < 0.05.

## 3. Results

### 3.1. Clinical Characteristics

The demographic and clinical characteristics of the study participants are shown in Table 1. Among the 912 patients, postoperative delirium was observed in 185 (20.3%). Patients with delirium were younger (years, 46.7 ± 15.7 vs. 52.8 ± 14.9, *p* < 0.001), had a higher proportion of males (78.9% vs. 68.4%, *p* = 0.005), and had a greater height (cm, 170.3 ± 8.9 vs. 167.7 ± 9.1, *p* = 0.001) and weight (kg, 76.2 ± 15.2 vs. 72.7 ± 15.6, *p* = 0.007), but there was no difference in BMI (kg/m^2^, 26.2 ± 4.3 vs. 25.7 ± 4.5, *p* = 0.237). Additionally, the delirium group had lower rates of HTN (23.8% vs. 36.9%, *p* = 0.001) and DM (8.1% vs. 13.8%, *p* = 0.039).

Regarding surgical characteristics, the delirium group had a higher rate of midazolam use than the non-delirium group (22.7% vs. 9.2%, *p* < 0.001). In terms of the type of surgery, fewer patients in the delirium group had undergone general surgery (2.2% vs. 19.5%, *p* < 0.001), obstetrics and gynecology (0.5% vs. 4.5%, *p* = 0.010), orthopedic surgery (4.9% vs. 13.3%, *p* = 0.001), or urology (0.5% vs. 10.6%, *p* < 0.001) procedures, but more patients had undergone ear, nose, and throat surgeries (75.7% vs. 36.7%, *p* < 0.001). The delirium group also had longer surgery durations (minutes, 124.5 ± 96.9 vs. 102.9 ± 83.7, *p* = 0.003) and a higher proportion of emergency surgeries (10.3% vs. 6.1%, *p* = 0.049).

The laboratory results indicated that the delirium group had higher rates of hypoalbuminemia (5.7% vs. 1.0%, *p* = 0.001) and hyponatremia (5.8% vs. 1.8%, *p* = 0.021). Other demographic, surgical, and laboratory variables that did not show statistically significant differences between the delirium and non-delirium groups are shown in Table 1.

### 3.2. Sleep Characteristics

The sleep variables associated with delirium are summarized in Table 2. There were no significant differences in sleep latency (minutes, 16.2 ± 38.9 vs. 12.5 ± 22.6, *p* = 0.214) or efficiency (%, 82.4 ± 15.8 vs. 81.9 ± 14.1, *p* = 0.686) between the groups. However, the delirium group had fewer REM episodes compared with the non-delirium group (5.9 ± 4.8 vs. 6.9 ± 5.3, *p* = 0.019). Additionally, the AHI was higher in the delirium group (42.6 ± 27.7 vs. 38.1 ± 27.1, *p* = 0.049). The delirium group also had fewer PLMar (1.2 ± 4.6 vs. 2.8 ± 12.2, *p* = 0.007). The sleep questionnaires, including the PSQI (*p* = 0.730), ISI (*p* = 0.841), ESS (*p* = 0.097), STOP-Bang (*p* = 0.801), and Berlin questionnaire (*p* = 0.770), did not show any statistically significant differences between the delirium and non-delirium groups.

### 3.3. Performances of Machine Learning Models

Table 3 shows the performance of six machine learning models (Logistic Regression, Random Forest, XGBoost, LightGBM, SVM, and ANN) developed using the training set and validated on an independent test set. The model with the highest performance was XGBoost (AUROC = 0.8351), followed by LightGBM (AUROC = 0.8209), ANN (AUROC = 0.7959), Logistic Regression (AUROC = 0.7896), Random Forest (AUROC = 0.7317), and SVM (AUROC = 0.5030).

**Figure 2:**
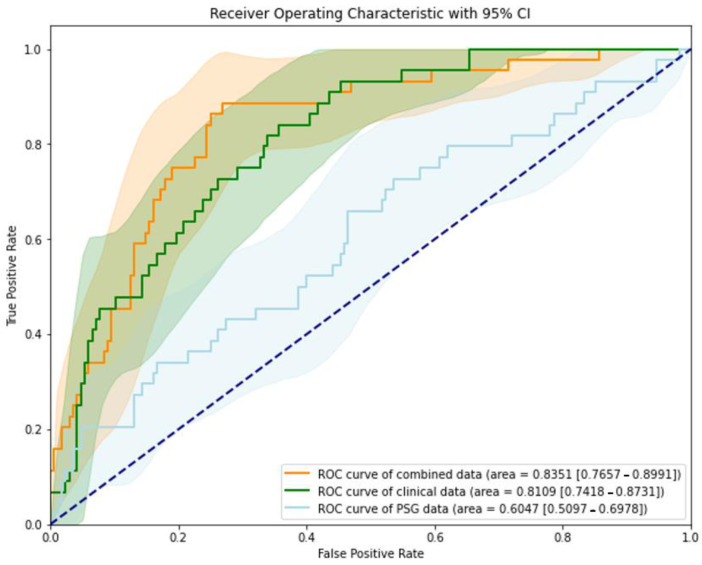
Receiver operating characteristic (ROC) curves for XGBoost model using clinical variables, polysomnography variables, and combined variables in predicting postoperative delirium.

When the best-performing XGBoost model was created using only clinical variables, the AUROC was 0.8109 (95% confidence interval [CI], 0.7657–0.8991). Using only sleep variables, the AUROC was 0.6047 (95% CI, 0.5097–0.6978). Combining both clinical and sleep variables resulted in an increased AUROC of 0.8351 (95% CI, 0.7657–0.8991). Figure 2 shows the receiver operating characteristic curve of this model, demonstrating that the combined model of clinical and sleep variables outperformed models that predict using only clinical or sleep variables.

### 3.4. Feature Importances

**Figure 3:**
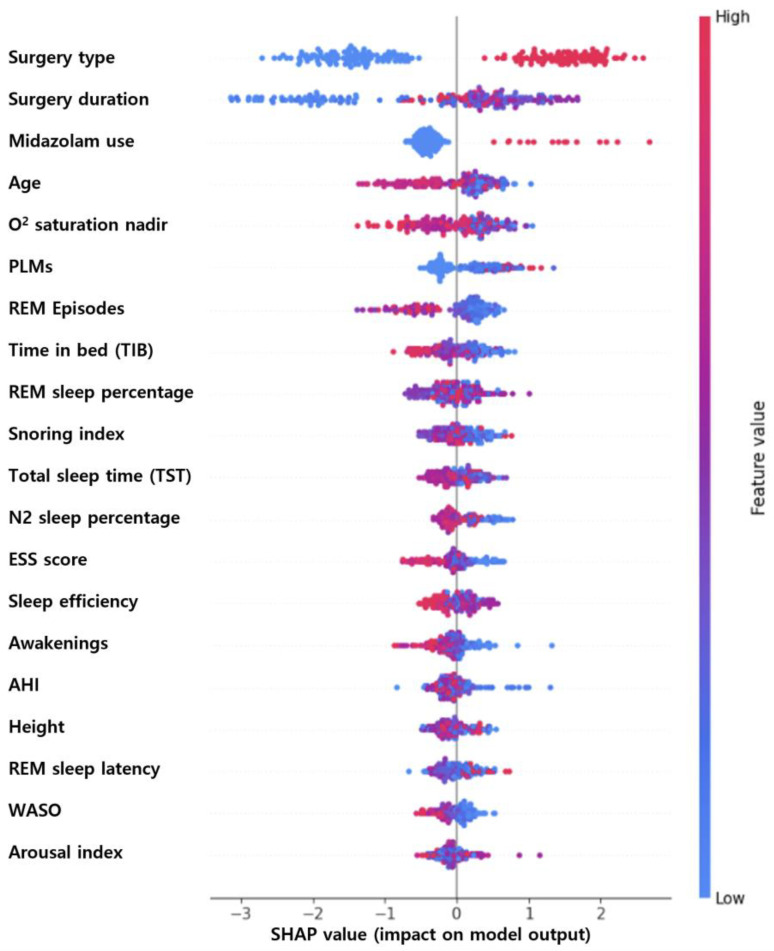
Summary plot of Shapley additive explanations (SHAP) values for the extreme gradient-boosting (XGBoost) model.

Figure 3 illustrates feature importance using SHAP values. Among the top ten features, the most important clinical variables were surgery type, operation duration, midazolam use, and age. The significant sleep-related variables included minimum O2 saturation, PLMs, REM episodes, TIB, REM percentage, and snoring.

## 4. Discussion

The key findings of this study were as follows: (1) the clinical variables associated with delirium were younger age, male sex, longer surgery duration, midazolam use, type of surgery, hyponatremia, and hypoalbuminemia; (2) the sleep variables associated with delirium included the AHI, REM episodes, and PLMar; and (3) among the machine learning models that combined clinical and sleep variables, the XGBoost model demonstrated the best performance. The addition of sleep variables to the clinical model resulted in an additional improvement in performance. Furthermore, variables previously associated with delirium played a significant role as important explanatory features in the model.

In previous studies, older age was a common clinical variable associated with delirium [2]. However, in our research, the overall study population was younger than those in previous studies, and we observed a higher incidence of delirium in younger patients compared with older ones. This discrepancy may be due to our definition of delirium, which included cases that required management, such as the addition of antipsychotic medications or psychiatric consultations, and predominantly hyperactive delirium. This type of delirium is more frequently observed in younger patients, which could explain the lower incidence in the older age group and the higher prevalence of HTN and DM in the study [27]. Consistent with previous research, our study also identified emergency surgery, longer surgery duration, and midazolam use as significant risk factors for delirium, consistent with established surgical risk factors [2,28,29]. While propofol was used in the majority of patients in this study, midazolam was administered to a subset of patients who required additional sedation. The use of midazolam may not only increase the risk of delirium through its promotion of inhibitory neurotransmission but also pose a secondary risk due to the disruption of sleep patterns, which can further contribute to the development of delirium. Considering the findings from in vivo and in vitro models suggesting that exposure to anesthetic agents may increase the risk of Alzheimer’s disease, the mechanisms underlying the role of anesthesia in postoperative cognitive changes need to be elucidated [30]. Additionally, our findings confirmed that laboratory results indicating the deterioration of general conditions, such as hyponatremia and hypoalbuminemia, were also associated with delirium.

In terms of sleep variables, the AHI was higher in the delirium group. A previous study also found that a higher AHI in preoperative PSG is associated with an increased risk of delirium in patients undergoing elective cardiac surgery [17]. These results suggest that, similar to cognitive decline, repetitive hypoxemia in patients with obstructive sleep apnea may cause oxidative stress and reperfusion injury, which could contribute to the development of delirium [31]. Additionally, we observed a reduction in the number of REM episodes in the delirium group. Previous studies have identified a relationship between delirium and reduced REM sleep, suggesting that pathways related to REM sleep, particularly serotonergic-mediated pathways, may be involved in psychiatric manifestations, potentially contributing to the occurrence of delirium [32,33].

In terms of sleep-questionnaire responses, previous studies have reported that higher PSQI scores, indicating poor sleep quality, are associated with an increased risk of delirium [13,14]. In this study, however, the results from questionnaires such as the PSQI, ISI, ESS, STOP-Bang, and Berlin Questionnaire did not show a significant association with delirium. Nevertheless, considering the impact of these self-report scores on cognitive decline in older adults, further research appears to be necessary [34].

In this study, the delirium prediction model developed by combining clinical data and sleep variables of surgical patients demonstrated a clinically applicable performance level with an AUROC of 0.84. This performance was comparable to that of existing models developed for patients in the intensive care unit (ICU) [35]. The model from this study is expected to play a particularly important role in clinical settings, especially in predicting hyperactive delirium, which typically requires clinical intervention [36]. The SHAP value rankings indicated that well-known clinical variables were more important than sleep variables. However, the model that combined clinical and sleep variables showed superior performance, suggesting that incorporating sleep variables is necessary to identify postoperative delirium in individual patients accurately.

This study had some limitations. First, the study was conducted retrospectively, with PSG results collected for 5 years before and after surgery. The time difference between the surgery and the PSG was not included in the analysis, which may have affected the accuracy of the machine learning model that relied on sleep-related variables. Furthermore, due to the nature of a one-night PSG, less consistent variables such as sleep architecture and more consistent variables such as sleep apnea were treated similarly. The significant night-to-night variability in PSG results may have further compounded this issue [37]. Second, instead of using standardized tools such as the Diagnostic and Statistical Manual of Mental Disorders or the Confusion Assessment Method to assess delirium, we used indirect methods such as diagnostic codes and medication use. This approach might have introduced a bias toward including more cases of hyperactive delirium and could also explain why the delirium patient group in this study was younger than expected. Third, because this study was conducted solely in the Korean population, it is unclear whether the findings are applicable to non-Asian populations.

This study had several strengths. First, it pioneered the creation of an AI model that integrates clinical variables and sleep features identified from PSG to predict postoperative delirium. This novel approach allows for a comprehensive evaluation of patients at high risk of delirium and enables the identification of influential factors, aiding in better prevention and management strategies. Second, the study employed a robust and objective assessment of sleep using PSG along with various sleep questionnaires. This study demonstrated that sleep-related factors offer additional predictive value for delirium by analyzing the performance and limitations of models utilizing clinical and PSG-derived sleep variables. When available in surgical patients, PSG data can offer additional valuable insights into the risk of delirium.

## 5. Conclusions

The AI model, which incorporated clinical variables with sleep patterns, showed consistent efficacy in predicting postoperative delirium on an individual basis. Notably, sleep-related factors contributed additional predictive power when combined with clinical data. This study was one of the first to explore the integration of sleep patterns with clinical data using machine learning for delirium prediction, suggesting that AI-assisted care could enhance predictive accuracy and help tailor interventions, ultimately reducing the risk of postoperative delirium.

## Figures and Tables

**Table 1:** Demographic and clinical characteristics of patients in the study.

	Delirium (+) (*n* = 185)	Delirium (−) (*n* = 727)	*p* Value
Age (years)	46.7 ± 15.7	52.8 ± 14.9	<0.001
Male sex, *n* (%)	146 (78.9)	497 (68.4)	0.005
Height (cm)	170.3 ± 8.9	167.7 ± 9.1	0.001
Weight (kg)	76.2 ± 15.2	72.7 ± 15.6	0.007
Body mass index (kg/m^2^)	26.2 ± 4.3	25.7 ± 4.5	0.237
Underlying comorbidities			
HTN, *n* (%)	44 (23.8)	268 (36.9)	0.001
DM, *n* (%)	15 (8.1)	100 (13.8)	0.039
Cardiac disease, *n* (%)	8 (4.3)	53 (7.3)	0.149
Brain disease, *n* (%)	6 (3.2)	35 (4.8)	0.357
ASA III–IV, *n* (%)	54 (29.2)	247 (34.0)	0.216
Anesthetic agents			
Midazolam, *n* (%)	42 (22.7)	67 (9.2)	<0.001
Propofol, *n* (%)	152 (82.2)	619 (85.1)	0.316
Operation type			
CS, *n* (%)	16 (8.6)	55 (7.6)	0.623
GS, *n* (%)	4 (2.2)	142 (19.5)	<0.001
NS, *n* (%)	14 (7.6)	56 (7.7)	0.951
OBGY, *n* (%)	1 (0.5)	33 (4.5)	0.010
OS, *n* (%)	9 (4.9)	97 (13.3)	0.001
URO, *n* (%)	1 (0.5)	77 (10.6)	<0.001
ENT, *n* (%)	140 (75.7)	267 (36.7)	<0.001
Surgery duration (min)	124.5 ± 96.9	102.9 ± 83.7	0.003
Emergency surgery (%)	19 (10.3)	44 (6.1)	0.043
Lab results *			
Anemia, *n* (%)	19/124 (15.3)	78/503 (15.5)	0.959
Thrombocytopenia, *n* (%)	6/124 (4.8)	27/503 (5.4)	0.813
Hypoalbuminemia, *n* (%)	7/123 (5.7)	5/511 (1.0)	0.001
AST/ALT elevation, *n* (%)	28/123 (22.8)	92/517 (17.8)	0.204
Cr elevation, *n* (%)	6/123 (4.9)	31/510 (6.1)	0.610
Hyponatremia ^†^, *n* (%)	7/121 (5.8)	9/502 (1.8)	0.021
Hypokalemia ^†^, n (%)	0/121 (0.0)	3/503 (0.6)	1.000

* Laboratory results presented in the table reflect only observed measurements, excluding missing data. The denominator for each value represents the number of patients for whom data were available. ^†^ Fisher’s exact test was used. HTN, hypertension; DM, diabetes mellitus; ASA, American Society of Anesthesiologists class; CS, cardiac surgery; GS, general surgery; NS, neurosurgery; OBGY, obstetrics and gynecology surgery; OS, orthopedic surgery; URO, urological surgery; ENT, ear, nose, and throat surgery; AST, aspartate aminotransferase; ALT, alanine aminotransferase; Cr, creatinine.

**Table 2:** Sleep characteristics and their association with postoperative delirium risk.

	Delirium (+) (*n* = 185)	Delirium (−) (*n* = 727)	*P* Value
Sleep latency (min)	16.2 ± 38.9	12.5 ± 22.6	0.214
TIB (min)	434.3 ± 40.2	439.6 ± 39.5	0.104
TST (min)	355.4 ± 64.5	357.7 ± 57.1	0.646
WASO (min)	60.0 ± 55.7	67.1 ± 58.5	0.136
Sleep efficiency (%)	82.4 ± 15.8	81.9 ± 14.1	0.686
N1 stage (%)	38.9 ± 18.6	37.3 ± 18.0	0.294
N2 stage (%)	45.7 ± 16.2	47.2 ± 15.4	0.247
N3 stage (%)	0.6 ± 2.2	0.4 ± 1.9	0.196
REM stage (%)	14.8 ± 6.5	15.1 ± 6.5	0.514
REM episodes (*n*)	5.9 ± 4.8	6.9 ± 5.3	0.019
REM latency (min)	142.7 ± 89.4	147.0 ± 87.5	0.559
Awakenings (*n*)	29.9 ± 20.0	32.9 ± 21.2	0.088
Arousal index (/h)	42.7 ± 20.5	40.4 ± 20.3	0.167
AHI (/h)	42.6 ± 27.7	38.1 ± 27.1	0.049
OSA classification			
No, *n* (%)	8 (4.3)	59 (8.1)	0.161
Mild, *n* (%)	30 (16.2)	106 (14.6)	
Moderate, *n* (%)	34 (18.4)	162 (22.3)	
Severe, *n* (%)	113 (61.1)	400 (55.0)	
O2 min (%)	81.1 ± 8.2	82.2 ± 8.2	0.098
Snoring index (/h)	219.9 ±159.6	231.7 ± 161.2	0.374
PLM index (/h)	7.5 ± 21.8	8.0 ± 18.7	0.742
PLMar index (/h)	1.2 ± 4.6	2.8 ± 12.2	0.007
Sleep questionnaire *			
PSQI	7 (5.5–11.5)	8 (5–11)	0.730
ISI	11 (7–17)	11 (7–16)	0.841
ESS	8 (4–12)	7 (4–11)	0.097
STOP-Bang	4 (3–6)	4 (3–5)	0.801
Berlin questionnaire (high, %)	130 (71.0)	500 (69.9)	0.770

* Analysis of continuous variables from sleep questionnaires was conducted using the Mann–Whitney U test as the data did not conform to a normal distribution. TIB, time in bed; TST, total sleep time; REM, rapid eye movement sleep; AHI, apnea–hypopnea index; OSA, obstructive sleep apnea; PLMs, periodic limb movements; PLMar, periodic limb movements with arousal; PSQI, Pittsburgh Sleep Quality Index; ISI, insomnia severity index; ESS, Epworth Sleepiness Scale.

**Table 3:** Performances of machine learning models on the test set.

Models	Accuracy	Precision	Recall	F1-Score	AUROC (95% CI)
Logistic Regression	0.8113	0.6429	0.2045	0.3103	0.7884 (0.7157–0.8571)
Random Forest	0.7972	0.6667	0.0455	0.0851	0.7908 (0.7160–0.8574)
XGBoost	0.7783	0.4348	0.2273	0.2985	0.8037 (0.7279–0.8658)
Light GBM	0.7972	0.5238	0.2500	0.3385	0.7980 (0.7235–0.8663)
SVM	0.7972	1.0000	0.0227	0.0444	0.7610 (0.6868–0.8254)
ANN	0.8113	0.7858	0.8113	0.7857	0.7959 (0.7120–0.8650)

AUROC, area under the receiver operating characteristic curve; XGBoost, extreme gradient boosting; LightGBM, light gradient-boosting machine; SVM, support vector machine; ANN, artificial neural network.

## Data Availability

Anonymized data relevant to this study will be shared upon request with a qualified investigator, pending appropriate Institutional Review Board approval.
